# The role of topical simvastatin on bone regeneration: A systematic review

**DOI:** 10.4317/jced.51415

**Published:** 2014-07-01

**Authors:** Javier Montero, Guillermo Manzano, Alberto Albaladejo

**Affiliations:** 1 DDS. PhD, Tenured lecturer in Prosthodontics. Department of Surgery, Faculty of Medicine, University of Salamanca (USAL), Salamanca, Spain; 2 DDS, Postgraduate Student. Master in Dental Sciences. University of Salamanca (USAL), Salamanca, Spain; 3 DDS. PhD, Tenured lecturer in Orthodontics. Department of Surgery, Faculty of Medicine, University of Salamanca (USAL), Salamanca, Spain

## Abstract

Objectives: The aim of this systematic review was to summarize the results concerning the use of simvastatin for promoting bone regeneration and to discuss the level of scientific evidence supporting such findings. Material and Methods: A Pubmed search using “Simvastatin”[Mesh] AND “Bone Regeneration”[Mesh] as Boolean operators was constrained to the last 10 years and only included papers written in English. Results: Of the 41 relevant papers reviewed, most of them (76.2%) have been published in the last 5 years, and most of them address animal studies (66.6%) performed on rats or rabbits in extraoral regions. Only 4 randomized controlled trials (RCTs) assessed the role of topical simvastatin in periodontal patients. Conclusions: A large part of the evidence concerning the role of topical simvastatin on bone regeneration comes from animal studies (mainly rats) focusing on extraoral bone defects. Only the use of subgingival simvastatin after root scaling has been properly supported by RCT.

** Key words:**Simvastatin, bone regeneration, topical administration, osteogenesis, osteoinduction.

## Introduction

Since the popularization of dental implants, clinicians have found themselves in circumstances in which they are forced to place implants in areas where the quality, quantity and topography of bone substrate are clearly unfavorable. In addition, several regenerative strategies have been proposed with the aim of preserving the alveolar process after tooth extraction (1) or for augmenting bone support for dental implants (2). Thus, the demand for effective biomaterials for bone augmentation procedures is currently increasing owing to the disadvantages of using autogenous bone graft material, such as limited bone availability, additional morbidity at the donor site, the impossibility of storage, and the need for general anesthesia for extraoral donor sites (3). In light of this, researchers are now searching for bone graft materials with improved osteogenic properties, with the advantages of autologous bone grafts [osteogenesis, osteoinduction and osteoconduction], but without their disadvantages [donor site morbidity, difficulty of storage and maintenance, unlimited availability, etc.].

Alloplastic bone substitutes have been combined with different molecules for the efficient induction of bone formation. In 1965, Urist used hydrochloric acid for the demineralization of bone matrix and observed ectopic bone formation after the implantation of demineralized bone matrix within the soft tissues, calling this process bone formation by autoinduction (4). It was not until 1980 that the same author reported the identification in the rat organic bone matrix of an insoluble protein of low molecular weight called Bone Morphogenetic Protein [BMP] (5). However, the use of BMPs entails some problems such as their short life, storage and handling difficulties, inefficiency in the recognition of target cells, and high cost. This has hampered their popularization in bone regeneration procedures (6).

On searching for alternatives to the application of these exogenous genetically engineered proteins, some authors have suggested the topical use of drug compounds aimed at upregulating intrinsic bone growth factors. For example, some widely known pharmacologic compounds [such as bisphosphonates or statins] have recently been shown to upregulate bone growth through distinct and complex biochemical pathways (7,8). Along this line, in 1999 Mundy *et al*. (9) were the first authors to report that lovastatin and simvastatin stimulate bone regeneration when injected subcutaneously in mouse calvaria. Since then, a huge development of research focusing on clinical applications has occurred in traumatology (10), oral surgery (11), and periodontology (12).

Simvastatin is one of the most commonly prescribed drugs for the treatment of hypercholesterolemia because it prevents the synthesis of cholesterol (13). In addition to its lipid-lowering effects, simvastatin can also elicit some pleiotropic effects, leading to the modulation of the process of bone regeneration at the molecular and cellular levels (14). Simvastatin seems to play an important role in bone regeneration by participating directly in osteoblast activation [increasing BMP-2 expression]and in osteoclast inhibition (15), and also indirectly, by stimulating neovascularization [increasing the secretion of Vascular Endothelial Growth Factor] (16).

The biological feasibility and the biochemical pathways of topical simvastatin in bone regeneration have been reviewed recently (14), but the current level of evidence concerning regenerative applications of simvastatin has not been established in a systematic way.

## Objectives

The aim of this systematic review was to summarize the results on the use of simvastatin for promoting bone regeneration and to discuss the level of scientific evidence supporting such findings.

## Material and Methods 

In October 2013, we made a search in Pubmed using as keywords simvastatin and bone regeneration, obtaining 75 results. In addition, to limit the search results we used the following search strategy: “Simvastatin” [Mesh] and “Bone Regeneration” [Mesh], constraining the search to papers written only in English and published in the last 10 years obtaining 37 relevant hits. This search was complemented with a manual search of 4 relevant articles cited among the previously selected papers. The 41 papers were revised in depth, and their main findings have been summarized throughout the result and discussion sections, discussing current knowledge and noting future trends based on the level of evidence. The distribution of such 41 revised papers according to the date of publication and the study design is shown in  Table 1.

**Table 1:**
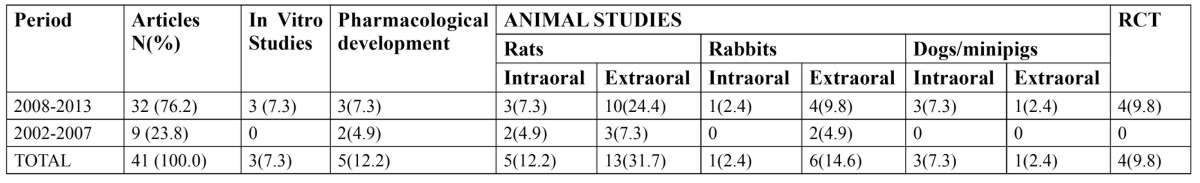
Distribution of the reviewed papers according to the study design (n=41).

## Results

Regarding study design (Table 1), it is to be noted that most of the revised papers [76.2%] have been published in the last 5 years, most of them involving animal studies [66.6%] performed on rats or rabbits in extraoral regions. To date, only one research team led by Pradeep has carried out 4 well-performed randomized clinical trials demonstrating that locally- administered simvastatin, versus placebo, significantly improves the clinical outcomes of scaling and root planing for treating mandibular buccal Class II furcation defects (17) and in patients with chronic periodontitis (18), even when they are smokers (19) or suffer from type 2 diabetes (20).

The *in vitro* studies have reported that simvastatin seems to stimulate bone formation significantly (21) and also aids in periodontal regeneration (22).

Studies focusing on pharmacological development have tested several biodegradable polymeric formulas for the local delivery of simvastatin, which is water-insoluble, such as a hydrogel of gelatin (23) or microspheres of polylactide-co-glycolide (24), although some carriers tested also seem to be able to release several bone-forming biomolecules simultaneously (25).

Regarding animal studies, it should be noted that most of them have been carried out in extraoral regions of rats ( Table 1) (26-33). The large majority of these studies reported favorable results concerning topical application of simvastatin, either injected alone (31) or in combination with biomaterials (28), or for coating implants (26) or covering acellular scaffolds (32). However, this osteogenic effect has also been observed when simvastatin was administered systemically by means of a 5mg/kg daily intraperitoneal (30) or oral dose (33).

In addition, most animal studies performed intraorally have reported good results for topical simvastatin administration in enhancing bone regeneration in rat mandibular defects (34) and periodontal lesions in rats [7,35], Beagles (36) and minipigs (37).

Nevertheless, other authors have reported unfavourable results after using simvastatin for bone formation. Pauly *et al*. (27) reported impaired integration of intramedulary titanium implants coated with simvastatin after 8 weeks of healing in rat femurs. In the same sense, Lima *et al*. (38) found a negative impact of combining simvastatin with demineralized bovine bone matrix for repairing calvarial defects in rats after 30 to 60 days of healing. In agreement with these studies on rats, in other studies performed on rabbits the authors failed to find any clear improvement on using topical simvastatin in osteoinductive activity for the short-term repair of nasal bone defects (15) or for enhancing mandibular distraction, even when applied both locally during the osteotomy phase, and systemically during the distraction osteogenesis period (11). Anbinder *et al*. also found that simvastatin administration, either orally or subcutaneously, did not improve bone repair for experimental tibial defects in rats (39). Rutledge *et al*. reported only minor effects of locally injected simvastatin for inducing new bone formation in the jaw bone of dogs (40).

## Discussion

Autologous bone is still the current gold-standard graft material for the treatment of bone defects (2). However, the need for a second surgical site, the limited supply of bone available, and the impossibility of storage have led to the development of different alternative bone substitutes. These biomaterials have also been combined with different bioactive agents as an appealing cost-effective way to promote bone formation. Statins [lovastatin, fluvastatin and simvastatin] have been demonstrated to modulate bone growth when applied locally (14). However, both clinicians and researchers are still in need of an updated evidence-based review of such promising regenerative applications in the field. This study summarizes the main findings of the investigations in which simvastatin was used to stimulate bone regeneration, and, to our knowledge, is the first systematic review addressing this topic. We focused only on simvastatin, since it is the statin most widely applied for this purpose, although other statins have also been investigated.

In light of our summarized results, the use of topical simvastatin for bone regeneration can be seen as a relatively recent research line, since most studies have been carried out in the last 5 years. The majority of the studies reviewed here were performed on small mammals [mainly rats but also rabbits], and generally in extraoral regions, reporting mainly favourable results for several bone surgical procedures. This shows that this research line is still in its initial stages. It should be taken into account that the different rates of bone turn-over in mammals, and throughout the bones of a given species, would hamper the extrapolation of such findings to the human mouth. Nevertheless 4 RCT were found with the search strategy used here. The fact that such well-designed trials were all performed at the same centre [Government Dental College and Research Institute of Bangalore, India] and by the same research team led by Pradeep could be a cause of concern. Those authors demonstrated a significant improvement in the clinical and radiographic parameters when a flowable gel of simvastatin [1.2mg/0.1ml] was injected at sites treated by scaling and root planing among different profiles of periodontal patients (17-20). Unfortunately no other promising application drawn from animal studies, such as the use of topical simvastatin for coating implants, or bone substitutes or acellular scaffolds, has been addressed in clinical trials. Therefore, the above data should encourage researchers and clinicians to replicate the formulation of the methylcellulose-based simvastatin gel described by Pradeep *et al*. (17-20) in order to check clinical outcomes in other settings or applications. It seems relatively simple to randomize and mask this intervention by a placebo gel, and it would not be very difficult to access the active principle since it is commercialized.

Caution should be exercised regarding the dosing of the simvastatin, since several authors have reported a dose-dependent inflammatory response (28). In animal studies, a 0.1-0.5 mg dose of simvastatin would be the optimal dose for stimulating maximum bone regeneration without inducing inflammation (37). According to Pradeep *et al*. (17-20), for humans the topical dose of simvastatin did not produce any complications or adverse reactions. Regarding the carrier used by Pradeep *et al*, methylcellulose was probably chosen for operational reasons [it is extensively used as a sustained-released vehicle for therapeutic formulations]; however, other polymeric carriers could also offer some advantages, such as a slower release of the molecules, or even multiple drug carriers (23-25).

Future efforts should be directed towards understanding the biological mechanisms underlying why a single topical application of simvastatin in a subgingival environment is able to act as an anti-inflammatory and regenerative agent of periodontal-tissues 6 to 9 months later, presumably affording a long-term effect (17-20). In light of several contradictory reports, it also remains unclear whether the oral consumption of statins has any significant effect on bone regeneration and periodontal health.

Long-term, multicenter, randomized, controlled clinical trials will be required to properly assess the effect of topical simvastatin in bone regeneration for many different indications.

## Conclusions

Most of the evidence regarding the role of topical simvastatin on bone regeneration, comes from animal studies [mainly rats], focusing on extraoral bone defects. However 4 well-designed RCT have reported significantly better clinical outcomes when a flowable gel of simvastatin [1.2mg/0.1ml] was injected at sites treated previously with scaling and root planing in periodontal patients. No other application has been properly assessed in humans.
